# Microstructure and Properties of Thermal Electrode Material Si_3_N_4_–MoSi_2_ Composite Ceramics

**DOI:** 10.3390/ma11060986

**Published:** 2018-06-11

**Authors:** Lichao Feng, Pengfei Guan, Xuemei Yu, Yiqiang He

**Affiliations:** 1 School of Mechanical and Ocean Engineering, Huaihai Institute of Technology, Lianyungang 222005, China; gpf543210@163.com (P.G.); yuxuemei_hit@126.com (X.Y.); gali_hit@126.com (Y.H.); 2 Marine Resources Development Institute of Jiangsu, Lianyungang 222001, China

**Keywords:** thermal electrode materials, hot pressing, microstructure, mechanical properties, electrical conductivity

## Abstract

With good high temperature and corrosive resistance performance, ceramic based composites can be used as promising materials to replace metal thermocouple materials. In this study, Si_3_N_4_–MoSi_2_ composites were prepared via hot pressing technology. X-ray diffraction (XRD), optical microscopy (OM), and scanning electron microscopy (SEM) were used to analyze the microstructure of the composites. The mechanical properties and electrical conductivity were tested. The results showed that the composites were composed of *β*-Si_3_N_4_, MoSi_2_, a small amount of Mo_5_Si_3_, and an amorphous glassy phase. The MoSi_2_ phase was evenly distributed in the matrix. The percolation network was formed with increasing MoSi_2_ content. The strength of the composites reached its maximum value when the MoSi_2_ content reached a critical point. The electrical conductivity behaved like a typical percolation phenomenon. The percolation threshold was about 30% to 45%.

## 1. Introduction

The thermocouple is the most widely used temperature testing device in many industrial areas [1,2,3]. To realize accurate temperature testing in a high temperature and corrosive environment, a corrosive and high temperature resistance tube has to be used to protect the metallic thermocouple material [4,5,6,7,8]. However, the application of the protection tube will reduce the stability and accuracy of the thermocouples due to the change of thermo-electrical potential resulting from the variation of the physical status of the metal electrode materials.

With increasing requirements from metallurgy, chemical, and aerospace engineering, the stable properties of thermocouple materials, which can be used in high temperature and corrosive environments, is still a big challenge in materials science, especially when they are used in molten salt electrolysis as the temperature testing device. Apart from the temperature range, the testing accuracy is another important factor that needs to be considered. As a result, a traditional metallic thermocouple is not able to satisfy the requirements when they are exposed to the above-mentioned working environments. Based on this condition, ceramic based composites, which have good high temperature and corrosive resistance performance, have been used as candidate materials to replace metallic thermocouple materials [9,10,11,12,13]. 

Disordered ceramic composites are composed of at least two types of ceramics, which combines the advantages of each component. Previous studies have demonstrated that Si_3_N_4_–MoSi_2_ composites have good mechanical, anti-oxidation, and corrosive resistance performances [14,15,16,17,18]. As a result, they are able to be used as candidate materials to fabricate thermocouple electrode materials. In this study, the mechanical and electrical properties and microstructure of Si_3_N_4_–MoSi_2_ composites were tested and analyzed to shed light on their potential application as new high temperature and corrosive resistance thermocouple materials. 

## 2. Experimental

**Figure 1:**
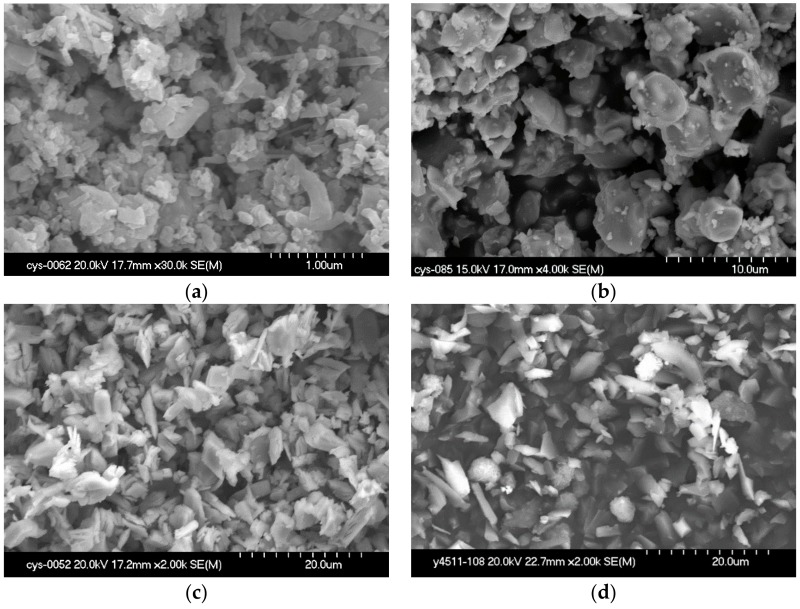
The SEM morphologies of the original powders. (**a**) *α*-Si_3_N_4_; (**b**) MoSi_2_; (**c**) Y_2_O_3_; (**d**) La_2_O_3_.

The Si_3_N_4_ powders were provided by Shinuorui Co. Ltd., Fuzhou, China and the MoSi_2_ powder was provided by Yantai Torch Special High Temperature Ceramics Co. Ltd., Yantai, China. The chemical contents are listed in Table 1, and the microstructures are depicted in Figure 1. All materials were used as received without further purification.

As can be seen from the chemical contents and microstructures, the purity of the *α*-Si_3_N_4_ was higher than 92% with an average size of 1 μm. The purity of La_2_O_3_, and MoSi_2_ was higher than 99% with size of 10 μm, and the purity of Y_2_O_3_ was higher than 99% with an average size of 15 μm, as shown in Table 1.

As listed in Table 2, the contents of MoSi_2_ were 30 wt %, 45 wt %, and 60 wt %, with 5 wt % of La_2_O_3_, 5 wt % of Y_2_O_3_ and the rest of Si_3_N_4_. Before the hot pressing process, the powders were ball-milled in dehydrated ethyl alcohol for 10 h, and subsequently dried in a vacuum furnace at 100 °C. After drying, the temperature was raised to 1750 °C with a heating rate of 30 °C/min, followed by hot pressing with 25 MPa and a 60 min soak in a graphite mold. The furnace chamber was purged with 0.5 atm of argon gas from the start of the hot pressing procedure. The relative densities of the prepared samples were tested according to the Archimedes method. The flexural strengths were tested through a three point bending method in a universal testing machine (Instron-5569, Instron, Norwood, CO, USA). The size of the samples were 3 × 4 × 36 mm^3^, and the flexural strength were calculated according to the following equation:

(1) $$\sigma =\frac{\;3FL}{2bh^{2}}$$

where *F* is the fracture loading; *L* is the space between the two points; and *b* and *h* are the width and thickness of the samples, respectively. The loading rate was 0.5 mm/min. The final results were the arithmetic average value of three samples. Before testing, the surfaces of the samples were polished to avoid stress concentration.

Thermal shock resistance is one of the most important properties of ceramic materials. It reflects the fracture resistance of a material with exposure to sharp temperature change conditions. In general, the thermal shock resistance can be characterized by the strength remaining rate after thermal shock testing, expressed as [19]:

(2) $$\eta_{rem}=\sigma_{T}/\sigma_{0}\times 100\%$$

where *η_rem_* is the flexural strength remaining rate; *σ_T_* is the flexural strength of the material experienced *n* time thermal shocking at temperature *T*; and *σ*_0_ is the original flexural strength. The samples for the remaining strength tests were air cooled at various temperatures 10 times. The final results were the arithmetic average value of three samples.

The phase structures were determined by X-ray diffraction pattern (XRD) on a Rigaku D/max-rA X-ray diffractometer with Cu Kα radiation (*λ* = 1.5406 Å) (Rigaku Corporation, Tokyo, Japan). The surface morphologies of the samples were observed by scanning electron microscopy with energy dispersive spectroscopy (SEM/EDS), performed on a Hitachi S-4700 scanning electron microscope (Hitachi Corporation, Tokyo, Japan). After that, the cross sectional optical microscopy was observed on Zeiss-MC80-DX (Zeiss, Oberkochen, Germany). 

## 3. Results and Discussion

### 3.1. XRD Results and Optical Microstructure

**Figure 2:**
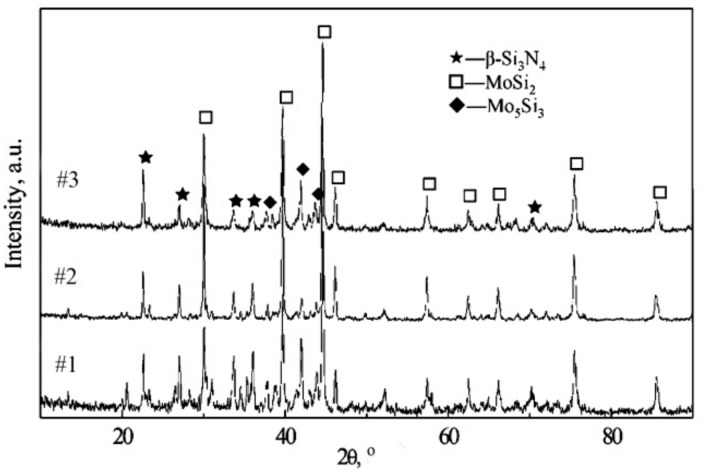
XRD pattern of the Si_3_N_4_–MoSi_2_ composites.

Figure 2 shows the XRD pattern of the Si_3_N_4_–MoSi_2_ composites. As demonstrated in this figure, the composites after hot compressing were mainly composed of the *β*-Si_3_N_4_ and MoSi_2_ phase. Neither Y nor La compounds were detected. Based on this result, it can be claimed that during the hot pressing process, the original *α*-Si_3_N_4_ changed to *β*-Si_3_N_4_, the sintering additives, Y_2_O_3_ and La_2_O_3_, reacted with the Si and O_2_ content and formed an amorphous glassy phase. In addition, small amounts of the Mo_5_Si_3_ phase could also be detected. 

**Figure 3:**
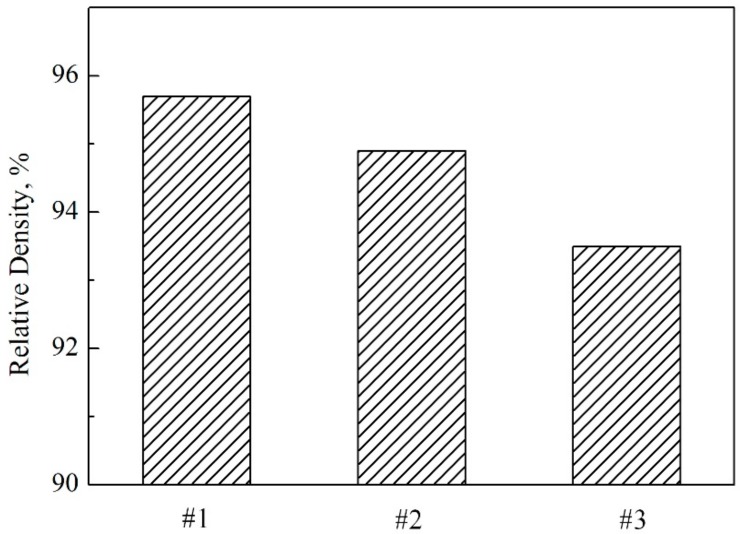
Relative density of the Si_3_N_4_–MoSi_2_ composites.

**Figure 4:**
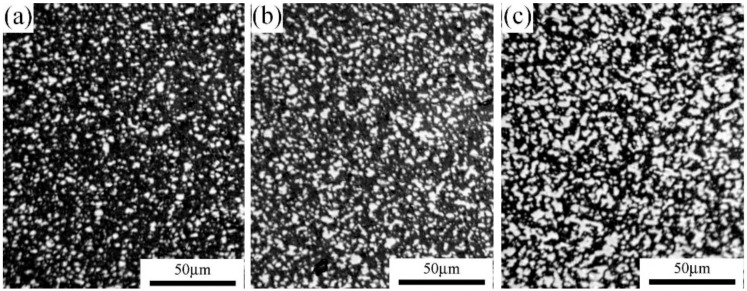
Optical microstructure of the Si_3_N_4_–MoSi_2_ composites of (**a**) Si_3_N_4_-30 wt % MoSi_2_, (**b**) Si_3_N_4_-45 wt % MoSi_2_, and (**c**) Si_3_N_4_-60 wt % MoSi_2_.

**Figure 5:**
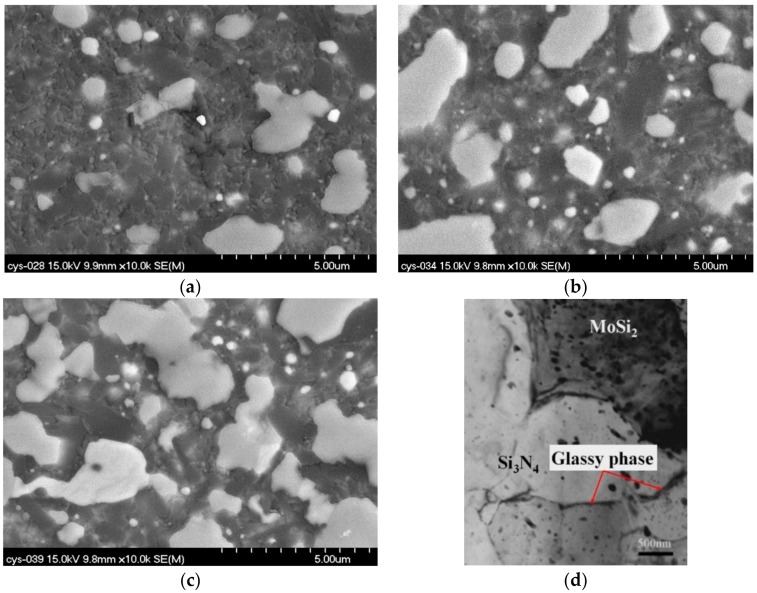
High magnification backscattering SEM of the Si_3_N_4_-MoSi_2_ composites (**a**) Si_3_N_4_-30 wt % MoSi_2_; (**b**) Si_3_N_4_-45 wt % MoSi_2_; (**c**) Si_3_N_4_-60 wt % MoSi_2_, and (**d**) TEM image.

The densities of the prepared samples are shown in Figure 3. As demonstrated in this figure, the densities of Si_3_N_4_-30 wt % MoSi_2_, Si_3_N_4_-45 wt % MoSi_2_, and Si_3_N_4_-60 wt % MoSi_2_ were 4.66 g/cm^3^, 4.31 g/cm^3^, and 3.99 g/cm^3^, respectively. After calculation, the relative densities of the samples were 93.5%, 94.9%, and 95.7%. As can be seen from these results, the samples were all well sintered with a relatively low porosity of less than 6.5%. Previous studies have demonstrated that the densification of Si_3_N_4_–MoSi_2_ composites is still a challenge without sintering additives [20,21,22,23]. It was found that with the addition of Y and La, the densification could be significantly improved [24,25]. Figure 4 and Figure 5 show the optical microscopy, backscattering SEM (BSE), and transmission electron microscopy (TEM) images of Si_3_N_4_-30 wt % MoSi_2_, Si_3_N_4_-45 wt % MoSi_2_, and Si_3_N_4_-60 wt % MoSi_2_, respectively. In the OM and BSE, the white areas are the MoSi_2_ phase and the dark areas are the Si_3_N_4_ phase. As can be seen in these figures, these two phases were evenly distributed, and the interfaces between the two components were clear and smooth. Little microcracks could be observed. The size of the Si_3_N_4_ was about 3–8 μm, and the size of the MoSi_2_ was about 3–5 μm with a uniaxial shape. In addition, the size of the Si_3_N_4_ slightly reduced with the increasing weight contents of MoSi_2_.

### 3.2. Flexural Strength

**Figure 6:**
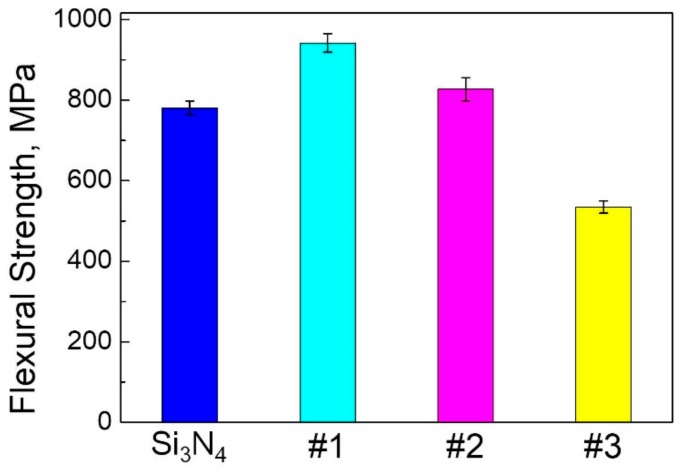
Flexural strengths of the Si_3_N_4_-MoSi_2_ composites.

Figure 6 shows the flexural strengths of the Si_3_N_4_–MoSi_2_ composites. As illustrated in this figure, the flexural strength of the composites reached 942 MPa, which was higher than that of the pure Si_3_N_4_ (781 MPa), when the filling content of MoSi_2_ reached 30 wt %. However, the flexural strength started to decrease with increasing MoSi_2_ content over 30 wt %. The flexural strength decreased to 535 MPa when the MoSi_2_ content reached 60 wt %. 

**Figure 7:**
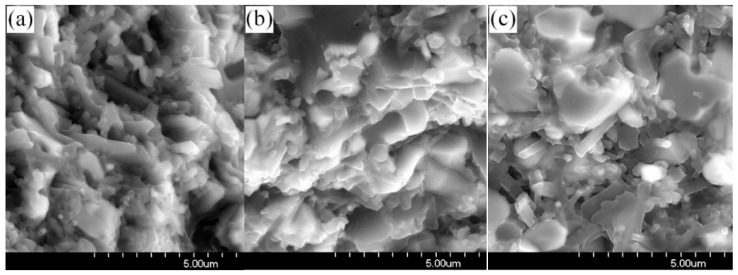
SEM microstructure of the fracture surfaces of Si_3_N_4_–MoSi_2_ composites of sample (**a**) Si_3_N_4_-30 wt % MoSi_2_; (**b**) Si_3_N_4_-45 wt % MoSi_2_; (**c**) Si_3_N_4_-60 wt % MoSi_2_.

Figure 7 shows the SEM morphology of the Si_3_N_4_–MoSi_2_ composites. As demonstrated in this figure, the fracture of the composites was a typical inter-granular fracture. The grains of the Si_3_N_4_ were rod shaped and the grains of MoSi_2_ were equiaxial. In sample #1, the grain size of MoSi_2_ was relatively small with a size of about 3–5 μm. With an increase of MoSi_2_ content, the grain size of MoSi_2_ kept increasing slightly, and small amounts of randomly distributed rod shaped Si_3_N_4_ crystals were observed among the MoSi_2_ crystals. It can be concluded that with the addition of MoSi_2_, the flexural strength of the composites will start to decrease when the content of MoSi_2_ is higher than a critical value due to the increase of porosity and residual stresses.

### 3.3. Thermal Shock Resistance

**Figure 8:**
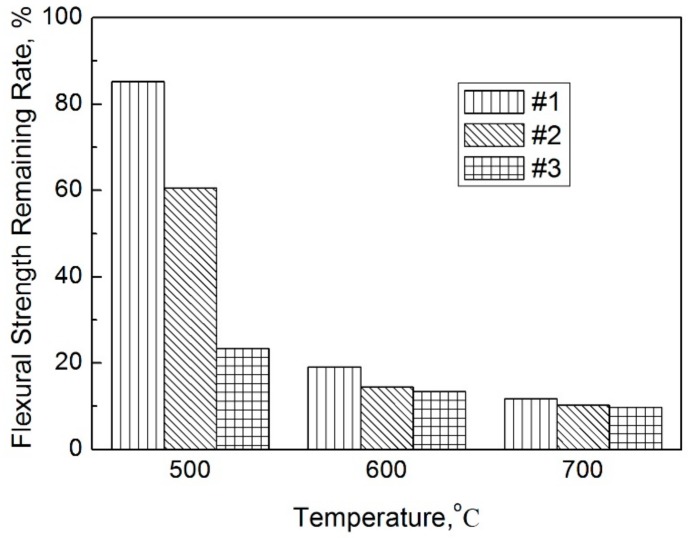
Flexural strength remaining rate of the Si_3_N_4_–MoSi_2_ composites at various temperatures.

Figure 8 presents the flexural strength remaining rate of the Si_3_N_4_–MoSi_2_ composites at temperatures of 500 °C, 600 °C, and 700 °C after thermal shock testing 10 times. As demonstrated in this figure, the flexural strength remaining rates were 85.2%, 60.5%, and 23.4% corresponding to samples #1, #2, and #3, respectively, when the thermal shock temperature was 500 °C. When the thermal shock testing temperature increased to 600 °C and 700 °C, the flexural strength remaining rate was lower than 20%. 

**Figure 9:**
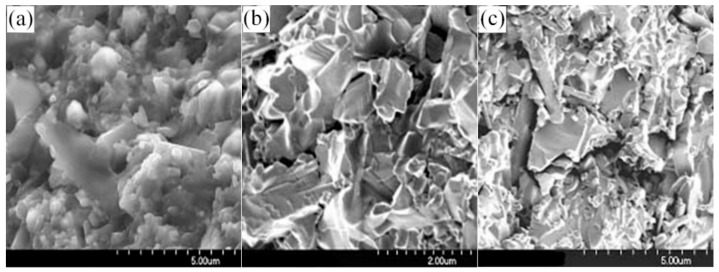
SEM microstructure of the fracture surfaces of the Si_3_N_4_–MoSi_2_ composite samples. (**a**) Before thermal shock, (**b**) thermal shock at 500 °C, and (**c**) thermal shock at 600 °C.

Figure 9 shows the SEM microstructure of the fracture surface of sample #1. As demonstrated in this figure, microcracks could be clearly observed when compared with the samples without thermal shock experience. In addition, the quantity and size of the microcracks increased with increasing thermal shock testing temperatures, which resulted from the internal stresses due to the thermal expansion mismatch. As a result, the flexural strengths decreased with the increasing internal stress and thermal mismatch of the two main constituents.

### 3.4. Electrical Conductivity

**Figure 10:**
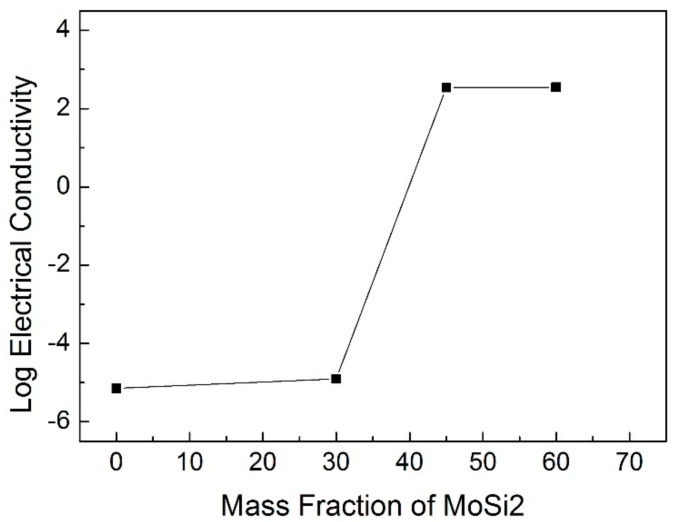
Electrical conductivity of the Si_3_N_4_–MoSi_2_ composites as a function of MoSi_2_ mass fraction.

Figure 10 illustrates the electrical conductivity of the Si_3_N_4_–MoSi_2_ composites as a function of filling content of MoSi_2_. As shown in this figure, the addition of the MoSi_2_ could considerably increase the electrical conductivity of the composites. When the content of MoSi_2_ reached 30 wt %, the electrical conductivity was 1.25 × 10^−5^ Ω^−1^·m^−1^; when the content increased to 45 wt %, the electrical conductivity increased seven orders of magnitude and reached 3.45 × 10^2^ Ω^−1^·m^−1^. After this value, the electrical conductivity increasing rate became flat. This phenomenon can be explained by the percolation theory [13]. Before the percolation threshold, which means that the content of MoSi_2_ is relatively low, the MoSi_2_ is randomly distributed in the matrix, but no percolation network can be formed. Under this condition, the electrical conductivity is mainly determined by the matrix material, and shows low electrical conductivity. When the MoSi_2_ content increased above the percolation threshold, the percolation network of MoSi_2_ formed, and as a result, the electrical conductivity was mainly contributed by the MoSi_2_ phase rather than the matrix, thus showing high electrical conductivity. In this study, it can be claimed that the percolation threshold is located between 30 wt % to 45 wt %. 

## 4. Conclusions

In summary, this study successfully prepared Si_3_N_4_–MoSi_2_ composites with hot pressing technology. The main constituents of the hot pressed Si_3_N_4_–MoSi_2_ composites were *β*-Si_3_N_4_ and MoSi_2_, and a small amount of the Mo_5_Si_3_ phase and glassy phase containing La and Y. The SEM morphology of the Si_3_N_4_–MoSi_2_ composites demonstrated that the fracture of the composites was a typical inter-granular fracture. The grains of the Si_3_N_4_ were rod shaped and the grains of MoSi_2_ were equiaxial. The strength of the Si_3_N_4_–MoSi_2_ composites decreased with increasing MoSi_2_ content. The flexural strength remaining rate of the Si_3_N_4_–MoSi_2_ composites at temperatures of 500 °C, 600 °C, and 700 °C after thermal shocking testing 10 times demonstrated that the flexural strength rates were 85.2%, 60.5%, and 23.4% corresponding to Si_3_N_4_-30 wt % MoSi_2_, Si_3_N_4_-45 wt % MoSi_2_, and Si_3_N_4_-60 wt % MoSi_2_, respectively, when the thermal shock temperature was 500 °C. When the thermal shock testing temperatures increased to 600 °C and 700 °C, the flexural strength remaining rates were lower than 20%.

## Figures and Tables

**Table 1:** Chemical contents of the original powders.

*α* Phase-Si_3_N_4_/wt %		MoSi_2_/wt %		La_2_O_3_/wt %		Y_2_O_3_/wt %	
purity	>92	purity	>99	purity	>99	purity	>99
N	>38.5	Si	>36.5	REO	>94.5	REO	>94.5
O	<1.5	O	<0.85	CaO	<0.15	CaO	<0.15
C	<0.1	Fe	<0.09	Fe_2_O_3_	<0.10	Fe_2_O_3_	<0.10
Fe	<200 ppm	C	<0.01	CeO_2_	<0.05	CeO_2_	<0.05

**Table 2:** Constituents of samples.

No.	Si_3_N_4_/wt %	MoSi_2_/wt %	La_2_O_3_/wt %	Y_2_O_3_/wt %
#1	60	30	5	5
#2	45	45	5	5
#3	30	60	5	5
